# Bending Forming Characteristics of CoCrFeMnNi High-Entropy Alloy Sheets Induced by Nanosecond Pulse Laser Irradiation

**DOI:** 10.3390/ma17153728

**Published:** 2024-07-27

**Authors:** Xinyu Tian, Chao Wang, Hongyang Zhang, Junfeng Gao, Hu Huang, Jiwang Yan

**Affiliations:** 1 Key Laboratory of CNC Equipment Reliability, Ministry of Education, School of Mechanical and Aerospace Engineering, Jilin University, Changchun 130022, China; xytian22@mails.jlu.edu.cn (X.T.); wangchao21@mails.jlu.edu.cn (C.W.); hyzhang19@mails.jlu.edu.cn (H.Z.); gaojf22@mails.jlu.edu.cn (J.G.); 2 Department of Mechanical Engineering, Faculty of Science and Technology, Keio University, Yokohama 223-8522, Japan; yan@mech.keio.ac.jp

**Keywords:** laser forming, high-entropy alloy, bending, hardness, temperature gradient mechanism

## Abstract

Laser bending forming, as a flexible and die-less forming approach, facilitates the three-dimensional shaping of sheets through the generation of thermal stress via laser-material interaction. In this study, the bending forming characteristics of CoCrFeMnNi high-entropy alloy sheets induced by nanosecond pulse laser irradiation were systematically investigated. The effects of parameters including laser power, scanning speed, number of scans, scanning interval, and sheet size on the bending angle, cross-sectional morphology, and hardness were studied in detail under both the laser single-line and multi-line scanning modes. The experimental results confirmed the effectiveness of nanosecond pulse laser irradiation for achieving accurate formation of CoCrFeMnNi sheets, with the successful fabrication of J, L, and U-shaped metal components. Apart from the forming ability, the cross-sectional hardness was significantly increased due to the grain refinement effect of nanosecond pulse laser irradiation. Furthermore, employing the laser single-line scanning mode enabled the effective rectification of overbending parts, showcasing complete recovery for small-angle overbending, and a remarkable 91% recovery for larger-angle overbending. This study provides an important basis for the bendability of CoCrFeMnNi sheets by laser forming and elucidates the evolution of the microstructure and mechanical properties in the bending region.

## 1. Introduction

High-entropy alloys (HEAs), consisting of five or more elements in equimolar or near equimolar proportions, break the traditional design idea of alloys dominated by one element and have become an emerging field of metallurgical research [1]. HEAs have stable phase structures, mainly including solid solution phases such as face-centered cubic (FCC), body-centered cubic (BCC), and hexagonal close-packed (HCP) phases, as well as biphasic structures, rather than complex intermetallic compounds. Due to their unique microstructures and tunable properties, HEAs exhibit a series of superior properties that are unmatched by conventional materials, such as excellent room-temperature strength, superior low-temperature mechanical properties, high wear resistance, and corrosion resistance [2,3]. Compared with typical BCC and HCP phase HEAs, FCC phase HEAs have excellent plastic deformation ability and higher impact toughness. The equiatomic CoCrFeMnNi HEA, as a typical representative of FCC single-phase HEAs, with good phase stability, has shown great potential for application due to its extremely high toughness and strength at low temperature [4,5].

With the rapid development of mechanical manufacturing technology and the increasing requirements for material properties in extreme environments, CoCrFeMnNi HEA is expected to be used in the fabrication of components for automobiles, precision machinery, and aerospace applications [6,7]. Traditional mechanical press forming and thermoforming processes play pivotal roles in producing high-volume sheets and shell parts due to their simplicity, high productivity, and consistent product quality. However, the application of these processes requires customized molds, resulting in long part production cycles, which make it difficult to adapt to modern market demands for low-cost, low-volume production. When fabricating low-strength metal parts, mechanical press forming often results in quality issues, such as surface indentation, and more rigid parts are susceptible to rebound problems [8,9]. For instance, Shao et al. focused on developing a rebound compensation method to mitigate rebound errors in the multi-point forming process of aluminum alloy double-bending components [10]. Laser forming exhibits distinct directionality and energy concentration. Upon interacting with materials, it generates a local thermal gradient, leading to non-uniform expansion of materials, which in turn induces thermal stresses to achieve true die-less and rebound-less forming [11,12]. Currently, it has become an effective solution for components with complex shapes and small-scale production requirements.

The laser bending forming mechanisms for sheets mainly depend on the geometry of the workpiece and the laser parameters. These mechanisms are categorized into the temperature gradient mechanism (TGM), buckling mechanism (BM), and upsetting mechanism (UM) [12,13,14,15]. TGM consistently causes the sheet to bend toward the laser source [16]. When a laser beam with a small spot diameter scans thicker sheets at a high scanning speed, the temperature on the top surface rises rapidly, while the lower surface remains cooler, resulting in a large temperature gradient [17]. The thermal expansion and yield strength reduction induced by laser irradiation leads to reverse buckling of the sheet. Since the free thermal expansion in the heated region is limited by the cooler materials, the heated region is pressurized, and a small amount of materials build-up occurs [18,19]. After the departure of the laser beam, materials located in the heated region of the top surface begin to cool and shrink. The build-up of materials caused by heating and compression is difficult to disappear due to the increase in yield stress [14]. The temperature of the lower surface rises due to heat conduction, leading to thermal expansion, which rapidly transforms a small reverse bending into significant forward bending [20,21,22].

The buckling mechanism also relies on a temperature gradient and is influenced by scanning speed. Typically, the workpiece is either thinner or has higher thermal conductivity [23,24]. This results in an essentially absent temperature gradient across the thickness of the sheet, which spreads outward from the scanning line. The generated compressive forces push against each other, leading to buckling instability, allowing the workpiece to bend towards or away from the laser [11,25]. The upsetting mechanism requires the workpiece to have a significant thickness or a high Young’s modulus [11]. When the laser scanning speed is low, the temperature gradient across the sheet thickness is extremely small [11]. Consequently, the bending moment is too large, resulting in insufficient plastic pressure to resist thermal expansion and bend the sheet. This causes localized thickening in the laser scanning region, shortening of the workpiece, and a change in Gaussian curvature [11,26,27].

Furthermore, the laser forming process offers advantages such as high flexibility, cleanliness, environmental friendliness, precise parameter control, high automation, and the ability to flexibly plan scanning paths [28,29]. Many researchers have explored various laser bending forming processes on conventional materials. For example, Abedinzadeh et al. investigated the laser forming process for unalloyed carbon steel sheets based on the orthogonal design of experimental methodology and discovered that the laser power and scanning speed had greater influences on the bending angle [30]. Yadav et al. applied the laser forming process to mild steel sheets and achieved a maximum bending angle of 22° after five scans [13]. Additionally, Khandai et al. investigated the laser forming of 0.5 mm AISI 304 stainless steel sheets. They utilized a real-time monitoring system to observe the process of laser forming with multi-pass in the laser modulation mode, and discovered that the bending mechanism changed from a temperature gradient mechanism to a buckling mechanism with the increase in scanning pass [31].

Previous research primarily focused on the bending forming of large sheets by using high-power lasers [13,30,31,32]. However, high laser energies usually result in larger heat-affected zones and low precision when forming miniature components. Some researchers have attempted to use small continuous lasers, such as laser markers, for micro-part bending forming. However, the limited laser power makes it challenging to achieve a large bending angle in a short time [33]. In contrast, nanosecond pulse laser, with short pulse width and high peak power, shows unique advantages in micro-component bending forming, enabling large-angle bending of sheets rapidly at low average power [31,34,35,36,37]. For example, in this study, a maximum bending angle of 90° was achieved for 0.5 mm CoCrFeMnNi high-entropy alloy sheets at an average power of no more than 40 W. Despite the theoretically advantageous characteristics of nanosecond pulse lasers, detailed studies on their application in the laser bending forming of emerging materials are still insufficient, and their specific feasibility and forming characteristics remain unclear.

Therefore, in this study, a nanosecond pulse laser was employed to irradiate the surface of CoCrFeMnNi HEA with the aim of investigating the feasibility of nanosecond pulse laser bending forming of CoCrFeMnNi sheets, and investigating the bending mechanism of the sheets as well as the mechanical properties of the bending region. The experimental results showed that laser irradiation achieved controllable bending forming of CoCrFeMnNi thin sheets, and also significantly improved the hardness of the bending region. These findings highlight the important potential and application value of laser forming technology for the manufacturing of complex components of CoCrFeMnNi HEA.

## 2. Materials and Methods

### 2.1. Materials and Experimental Procedures

CoCrFeMnNi HEA was employed as the experimental material. The length of the samples was 30 mm; the width covered five sizes, 25, 20, 15, 10, and 5 mm; the thickness included six types, 2.0, 1.5, 1.25, 1, 0.75, and 0.5 mm. The samples were cast by vacuum arc melting and remelted five times to ensure chemical homogeneity, and then obtained by the wire cutting process. Prior to the experiments, the samples were free from original deformation and were cleaned ultrasonically with anhydrous ethanol to remove oil and other contaminants.

**Figure 1:**
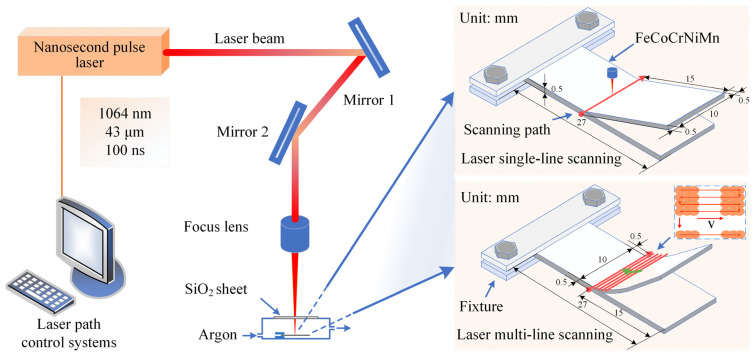
Schematic diagram of the laser irradiation system and scanning modes.

Then, one end of the sheet was clamped for 3 mm and placed in an atmospheric cavity. To ensure rapid surface cooling and prevent oxidation, argon gas with a pressure of 0.01 MPa was continuously introduced. A nanosecond fiber laser (SP-050P-A-EP-Z-F-Y, SPI, Rugby, UK) (laser wavelength: 1064 nm, pulse width: 100 ns, spot diameter: ~43 μm) was applied to irradiate the sample surfaces. The scanning line was positioned 15 mm from the free end of the sheet, and the scanning path extended beyond the sheet width by 1 mm to ensure that the laser power and scanning speed were in a stable state during the laser-forming process. Two laser scanning modes, laser single-line scanning mode and laser multi-line scanning mode, were employed to fabricate parts with straight and curved surface features. The operational principles of the laser irradiation system and the scanning modes are illustrated in Figure 1.

Based on pre-experimental results, the pulse width and laser repetition frequency were kept at 100 ns and 100 kHz, respectively. In order to deeply investigate the effects of key factors such as laser power, scanning speed, number of scans, and scanning interval on the bending angle during the laser forming process, a one-factor experimental method was employed here. The detailed parameters of the single-line scanning mode are given in Table 1, and the detailed parameter settings of the laser multi-line scanning mode are listed in Table 2. The corresponding bending angles were measured and analyzed using an optical microscope (DSX500, OLYMPUS, Shinjuku City, Japan).

### 2.2. Surface Characterization

The cross-section of the bending region was observed with a tungsten filament scanning electron microscope (SEM, JSM-IT500A, JEOL, Akishima, Japan). During SEM observation, the acceleration voltage, photo time, and working distance (WD) were 10.0 kV, 40.0 s, and 12.7 mm, respectively. The elemental distribution of laser-irradiated surfaces was detected by energy dispersive X-ray spectroscopy (EDS, EX-74600U4L2Q, JEOL, Akishima, Japan) at an accelerating voltage of 15 kV. The microstructure of the cross-section of the bending region was characterized by an electron backscatter diffraction detector (EBSD, C-nano, Oxford Instruments, Oxford, UK). The phase composition before and after laser irradiation was characterized by an X-ray diffractometer (XRD, D8 ADVANCE, BRUKER, Maryland, USA) with a scanning speed of 5°/min. The hardness of the bending region was tested with a nanoindentation instrument (DUH-211S, SHIMADZU, Kyoto, Japan) equipped with a pyramidal diamond indenter with an angle of 115° between the ridge and face (TOKYO DIAMOND Tools MFG. Co., Ltd., Meguro, Japan). The maximum load applied was 100 mN, with loading and unloading rates maintained at 10 mN/s. The holding time at the maximum load was set to 5 s to ensure consistent measurements. To ensure the reliability of the hardness measurements, 10 indentation tests were performed for each sample. The nanoindentation hardness was calculated using the Oliver–Pharr method.

## 3. Results and Discussion

### 3.1. Effects of Process Parameters on the Bending Angle

**Figure 2:**
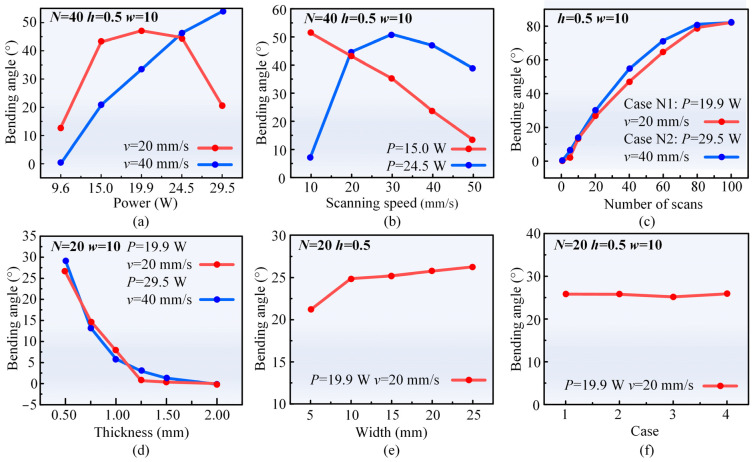
Effects of process parameters on the bending angle in the laser single-line scanning mode: (**a**) laser power, (**b**) scanning speed, (**c**) number of scans, (**d**) thickness, (**e**) sheet width, and (**f**) repeatability test.

Figure 2a,b demonstrate the effects of the laser power and scanning speed on the bending angle. At a scanning speed of 40 mm/s, the bending angle shows an increasing trend as the laser power increases. This phenomenon could be attributed to the increased temperature gradient in the thickness direction due to the increase in laser energy absorbed on the top surface of the sheet as the laser power increase, resulting in a corresponding increase in the bending angle. Moreover, the temperature rise causes an increase in the coefficient of thermal expansion of the CoCrFeMnNi HEA sheet and a decrease in yield strength [38,39], further contributing to the increase in the bending angle. When keeping the laser power at 15 W, the bending angle decreases with the increase in scanning speed. This phenomenon is attributed to the decrease in the overlap of adjacent pulse laser spots as the scanning speed increases, resulting in a decrease in energy absorption on the sheet surface and a decrease in the temperature gradient in the thickness direction. Meanwhile, the reduction in laser energy input leads to a decrease in the plastic deformation region and an increase in the yield strength of the materials [40,41], which eventually leads to a reduction in the bending angle.

However, at a scanning speed of 20 mm/s, the bending angle initially increases and then decreases with the increase in laser power. Similarly, when keeping the laser power at 24.5 W, the bending angle demonstrates an initial increase, followed by a decrease with the increase in scanning speed. Such variations arise from the presence of specific laser energy parameters required to achieve the maximum bending angle in bending forming. When the laser energy exceeds the optimum threshold, the temperature gradient decreases and the bending angle decreases consequently [39,42]. Notably, laser energy, being the primary factor influencing laser forming, exhibits a direct proportionality to laser power and an inverse proportionality to scanning speed [43,44].

Figure 2c illustrates the effects of the number of scans on the bending angle for two combinations of laser power and scanning speed. As the number of scans increases, the bending angles for both parameter combinations (Case N1 and Case N2) rise by 82° and 81°, respectively. However, the relationship between the number of scans and the bending angle is non-linear, with the slope of the curve gradually decreasing. Initially, when the temperature of the sheet is lower, the temperature gradient increases rapidly after laser irradiation, resulting in a significant increase in the bending angle. However, as the number of scans continues to increase, the cumulative heat input increases, and the cooling time is not short enough to bring the sample back to the initial temperature. The temperature gradient becomes smaller, and the increment of the bending angle decreases accordingly [40].

Figure 2d illustrates the effects of sheet thickness on the bending angle for two combinations of scanning speed and laser power. The bending angle decreases gradually to 0 degree as the sheet thickness increases from 0.5 mm to 2 mm. As the sheet thickness increases, the bending moment required to bend the sheet increases significantly, despite a slight increase in the temperature gradient. Consequently, more thermal stress is required to realize bending, resulting in a decreasing trend in the bending angle [45,46]. Figure 2e illustrates the effects of sheet width on the bending angle at a scanning speed of 20 mm/s and a laser power of 19.9 W. The bending angle increases with an increase in sheet width, and the incremental bending angle decreases when the sheet width exceeds 10 mm. Within a certain range, an increase in sheet width could enhance the rigid constraints imposed by the surrounding cold materials on the irradiated region, thereby increasing the plastic deformation of the irradiated region and increasing the bending angle. However, when the sheet width reaches a certain threshold, the rigid constraint increment gradually declines, leading to a leveling off of the change in bending angle [44]. The repeatability of the parameters is crucial for investigating the regularity of laser bending and forming. In the experiment, the laser power of 19.9 W, the scanning speed of 20 mm/s, and the number of scans of 20 are chosen as the experimental parameters for the repeatability test. Laser bending forming is repeated four times, and the bending angle is measured, as shown in Figure 2f. The average bending angle of the four tests is 25.97°, with a standard deviation of 0.33°. The low standard deviation of 0.33° demonstrates reliability and accuracy in assessing the regularity of the laser bending forming process.

**Figure 3:**
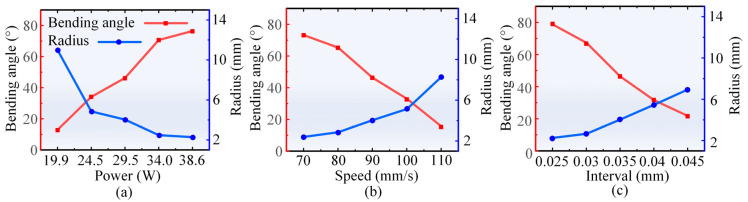
Effects of the process parameters on the forming shape in laser multi-line scanning mode: (**a**) laser power, (**b**) scanning speed, and (**c**) scanning interval.

Figure 3 illustrates the effects of the laser parameters on the forming shape in the laser multi-line scanning mode. As shown in Figure 3a, under a scanning speed of 90 mm/s and scanning interval of 0.035 mm, the bending angle gradually increases and the radius of the arc gradually decreases with the increase of the laser power. The bending process can be interpreted as the superposition effect of multiple single-pass laser scanning [47]. In Figure 3b, under a laser power of 29.5 W and a scanning interval of 0.035 mm, the bending angle gradually decreases and the radius of the arc gradually increases with the increase of the scanning speed. In Figure 3c, under a laser power of 29.5 W and a scanning speed of 90 mm/s, the bending angle gradually decreases and the radius of the arc gradually increases with the increase of scanning interval. The scanning interval equals the arc length divided by the number of scanning passes. Therefore, an increase in the scanning interval reduces the number of scanning passes, resulting in a decrease in the cumulative bending angle. In summary, the primary mechanism for bending CoCrFeMnNi sheets under the aforementioned parametric conditions is the temperature gradient mechanism.

### 3.2. Evolution of the Microstructure and Hardness

**Figure 4:**
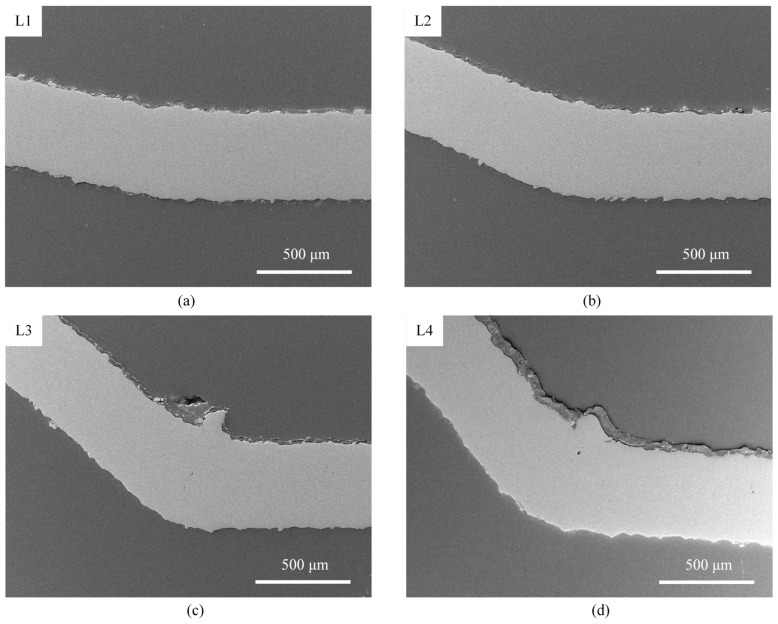
SEM morphologies of different laser-formed parts in laser single-line scanning mode: (**a**) Case L1, (**b**) Case L2, (**c**) Case L3, and (**d**) Case L4.

To investigate the microscopic morphology evolution during laser forming in the single-line scanning mode, cross-sections of the laser-formed parts under various parameters are observed. The detailed parameters for these laser-formed parts are provided in Table 3, and the corresponding microscopic morphologies are presented in Figure 4. Notably, the cross-sections of the bending region in Cases L1, L2, L3, and L4 exhibit no visible cracks. Additionally, a slight material buildup is observed in the bending region of sheets with a large bending angle. This phenomenon could be attributed to the temperature gradient mechanism, where the materials in the heated region are compressed and the yield strength is reduced due to the thermal effect induced by the pulse laser, leading to material buildups. However, during the subsequent cooling process, these buildups could not be recovered due to the increase in yield stress, ultimately leading to the buildup phenomenon observed in Figure 4 [27].

**Figure 5:**
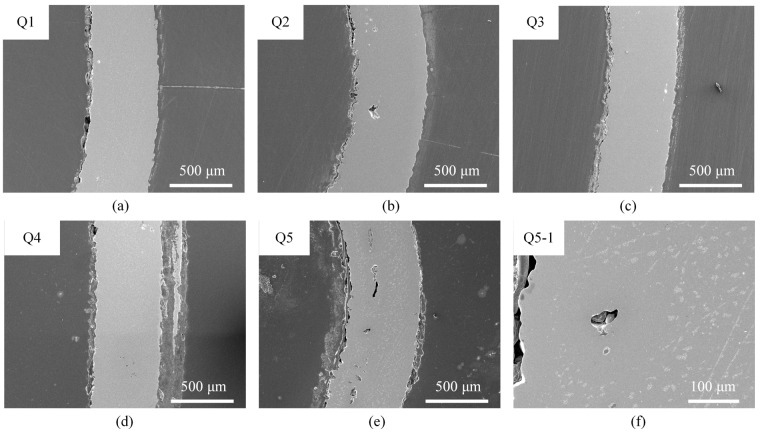
SEM morphologies of different laser-formed parts in laser multi-line scanning mode: (**a**) Case Q1, (**b**) Case Q2, (**c**) Case Q3, (**d**) Case Q4, and (**e**,**f**) Case Q5.

Additionally, five cross-sections of the laser-formed parts, each representing different parameter combinations, are selected to analyze the microscopic morphological evolution under the laser multi-line scanning mode. These parts, distinguished by varying laser power (Cases Q1, Q2, Q3) and scanning interval (Cases Q1, Q4, Q5), are detailed in Table 4. The corresponding microscopic morphologies are presented in Figure 5. Compared with the laser single-line scanning mode, the bending region in the multi-line scanning mode exhibits curved bending without obvious material buildup. The cross-sections of the bending region in the laser multi-line scanning mode show a similar microscopic morphology as in Figure 4, without any cracks. However, in the case of high power (Case Q5), a more obvious porosity phenomenon is observed. Therefore, a shorter scanning interval is utilized to achieve large-angle bending instead of higher laser power to ensure the quality of the bending region.

**Figure 6:**
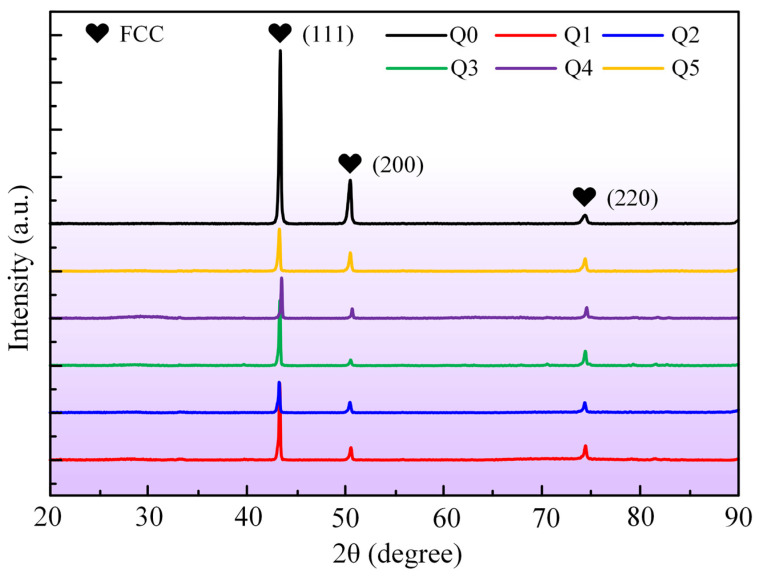
XRD patterns of the CoCrFeMnNi HEA sheets for Cases Q0, Q1, Q2, Q3, Q4, and Q5.

**Figure 7:**
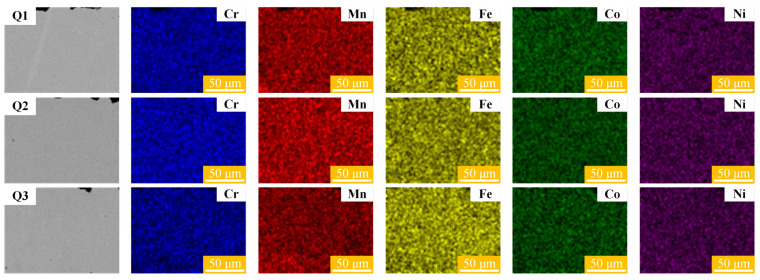
Elemental distribution of cross-sections in the bending region of Cases Q1, Q2, and Q3.

Further XRD analysis of the CoCrFeMnNi HEA sheets before and after laser bending formation is conducted to investigate the potential phase transformation induced by laser irradiation. Figure 6 illustrates the XRD patterns of the original sample (Q0) and the laser bending samples (Q1, Q2, Q3, Q4, and Q5). As illustrated in Figure 6, a single FCC peak appears on all samples. Compared to the original samples, the laser bending forming samples exhibit no phase transition, which confirms the stability of the CoCrFeMnNi HEA structure. To further investigate the chemical homogeneity of the material after laser irradiation, the cross-sections of the bending region are subjected to mechanical grinding, mechanical polishing, and ultrasonic cleaning sequentially. Subsequently, the elemental distribution is characterized, as shown in Figure 7. The results of EDS surface scanning demonstrate that the elements Cr, Mn, Fe, Co, and Ni in the cross-sections of the CoCrFeMnNi HEA are uniformly distributed. This indicates that the laser bending forming process does not lead to segregation or enrichment of these elements within the sheet. The laser bending forming process maintains the uniform elemental composition and maintains the chemical consistency of the CoCrFeMnNi HEA sheet.

**Figure 8:**
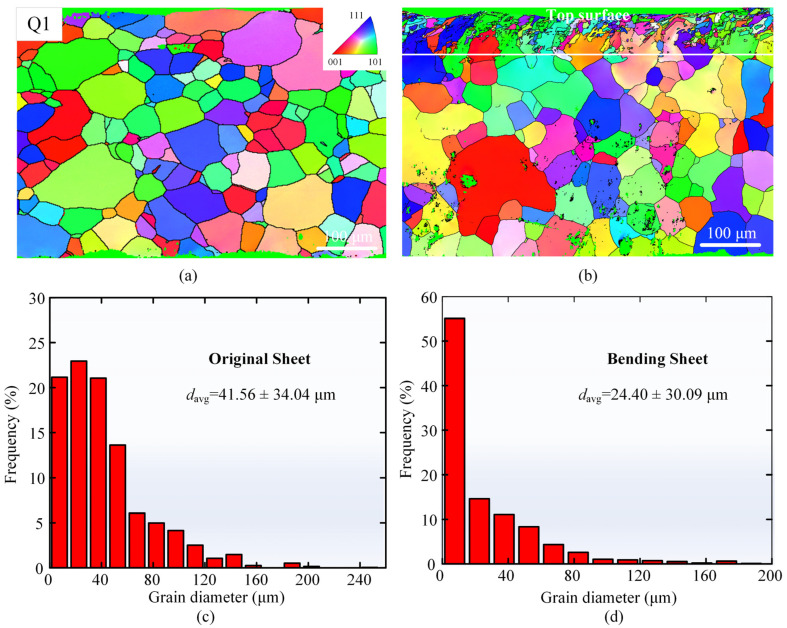
Cross-sectional EBSD results of (**a**) IPF of the original sheet, (**b**) IPF of Case Q1, (**c**) grain size distribution of the original sheet, and (**d**) grain size distribution of Case Q1.

Figure 8 shows the results of the EBSD analysis of the cross-sections of the original sheet and the bending region of Case Q1. The EBSD inverse polar figures (IPF) are shown in Figure 8a,b, and the IPF of case Q1 shows that the depth of grain refinement is approximately 81 μm. To investigate the variation of grain size over the entire thickness, the grain diameter size is analyzed comparatively. As depicted in Figure 8c,d, the average grain diameters in the cross-sections of the original sheet and the laser bending forming sample (Case Q1) are 41.56 ± 34.04 μm and 24.40 ± 30.09 μm, respectively. Significant grain refinement is observed in the cross-section of the laser bending forming sample, which could be attributed to the rapid heating/cooling effect induced by nanosecond pulse laser irradiation [41,48].

**Figure 9:**
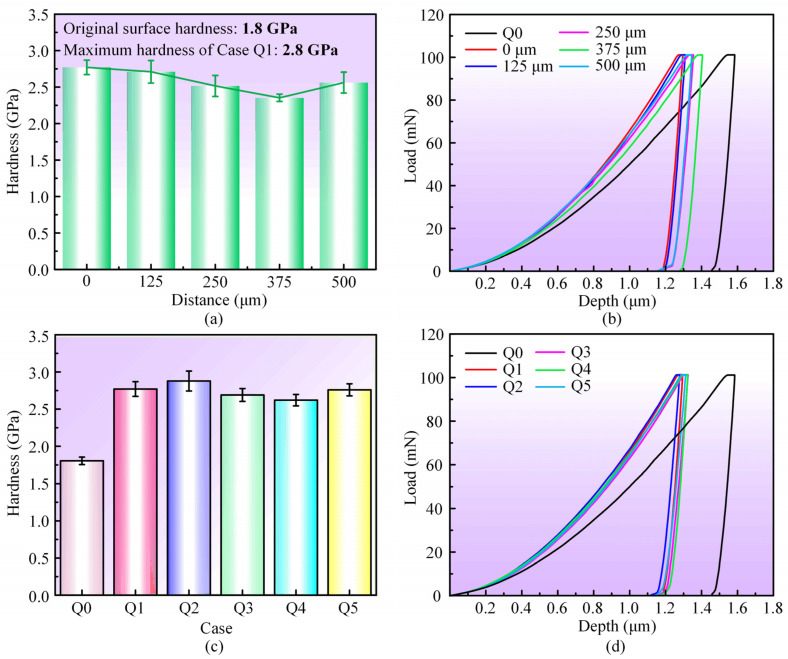
(**a**) Statistical results of hardness at various distances from the top surface for Case Q1, (**b**) load-depth curves at different depths for Case Q1, (**c**) statistical results of the highest hardness for all cases, and (**d**) load-depth curves for all cases.

Grain refinement typically leads to an improvement in surface mechanical properties, so nanoindentation tests are performed to assess the cross-sectional hardness in the bending region. In Figure 9a, the hardness variation of Case Q1 along the thickness direction of the sheet is illustrated. The hardness of the top surface for Case Q1 reaches 2.8 GPa, with a 55.6% improvement compared to the original surface (Case Q0) hardness of 1.8 GPa. As the distance from the top surface gradually increases, the hardness decreases, but it is still significantly higher than that of the original surface. Figure 9c illustrates the maximum hardness in the bending region of the different sheets. Case Q2 and Q5 sheets, characterized by smaller scanning intervals and larger laser power, respectively, exhibit a relatively larger hardness enhancement, while Case Q3 and Q4 show smaller hardness enhancement due to the utilization of larger scanning intervals and smaller laser power, respectively. Figure 9b,d show the load-depth curves, indicating a significant reduction in indentation depth in the laser bending region compared to the original surface, confirming the increase in hardness.

### 3.3. Application and Expansion of Laser Bending Forming

**Figure 10:**
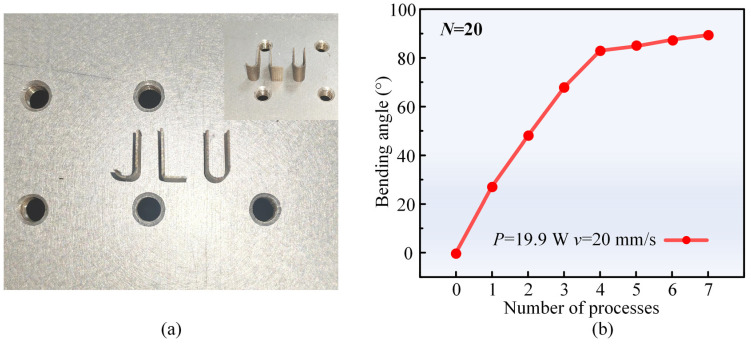
(**a**) Samples J, L, and U, and (**b**) effect of the number of processes on the bending angle.

Three miniature metal parts, denoted as J, L, and U, are fabricated to further explore the practicality of laser forming. The specific machining parameters for J, L, and U are detailed in Table 5, and the parts are shown in Figure 10a. The J- and U-shaped parts are formed through a composite process involving two 90° forming processes using the laser multi-line scanning mode. In the first operation, the scanning speed, laser power, and scanning interval are set to 90 mm/s, 31.8 W, and 0.03 mm, respectively. Subsequently, using a fixture to rotate the workpiece 90° clockwise, the scanning speed and interval are fixed, while the laser power is increased to 34.0 W for the second operation. Finally, a diamond wire cutter is employed to remove the excess material. For the L-shaped part, the laser process parameters are set as follows: laser power of 19.9 W, scanning speed of 20 mm/s, and number of scans of 20. This specific combination of laser power and scanning speed is chosen to maximize the bending angle while maintaining a fixed number of scans. Following each laser process, the sheet is cooled for 300 s. This approach is adopted due to the fact that although increasing the number of scans initially results in a significant increase in the bending angle beyond 80°, the bending angle increment slows down, making it challenging to reach 90°. Cooling for 300 s is helpful to prevent sheet melting during the process and to maintain a significant temperature gradient by lowering the sheet temperature before each laser process [12,49]. The variation in the bending angle with the number of processes is shown in Figure 10b.

**Figure 11:**
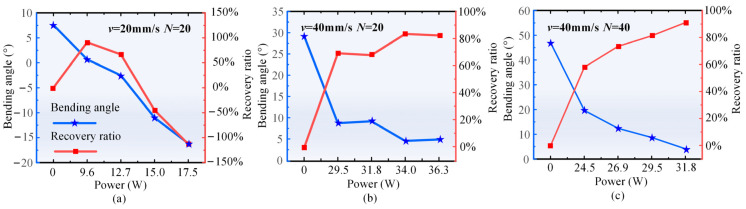
Effects of applying laser with various powers in the backward bending direction on the bending angle and the recovery ratio of pre-bending parts (when the horizontal coordinate is 0, the bending angle represents the pre-bending angle, and the recovery ratio is 0): (**a**) Case H1, (**b**) Case H2, and (**c**) Case H3.

The laser bending recovery technique is widely applied to solve common overbending problems in bending components [50]. Pre-bending parts with various angles are fabricated using a laser single-line scanning mode. Initially, these pre-bending parts are cooled to room temperature and then flipped and clamped on the fixture. Subsequently, employing the laser single-line scanning mode, with the number of scans and scanning speed held constant, different power lasers are applied to the pre-bending region of the parts. The aim is to adjust the deformation from a large angle to a small angle or even eliminate the angle entirely, thereby investigating the feasibility of the laser bending recovery technique. Table 6 provides detailed process parameters of the pre-bending parts, and Figure 11 illustrates the effects of different laser powers on the bending angle and recovery ratio of the pre-bending parts. The recovery ratio is defined as the ratio of the adjustment angle to the pre-bending angle. At a horizontal coordinate of 0, the bending angle corresponds to the pre-bending angle, and the recovery ratio is 0. Initially, identical laser process parameters as the pre-bending angle are applied for recovery across pre-bending parts with different angles. Subsequently, the laser power is gradually increased to explore the optimal recovery effect.

As shown in Figure 11a, for a pre-bending angle of 7.6°, 90% recovery can be achieved by applying the same laser parameters at the pre-bending position. With an appropriate increase in laser power, 100% recovery can be achieved; however, a further increase in laser power results in the recovered part re-bending towards the laser beam. The recovery effect for a pre-bending angle of 29.3° is depicted in Figure 11b, showing a 69% recovery with the same laser process parameters, while a maximum recovery of 81% is attained by increasing the laser power to 34.0 W. Nevertheless, a continual increase in laser power leads to excessive laser energy, diminishing the temperature gradient and reducing the bending recovery capability [39]. Figure 11c illustrates the recovery effect at a pre-bending angle of 47°, where a maximum recovery ratio of 91% is achieved by increasing the laser power to 31.8 W. These experimental findings validate the feasibility of laser bending recovery, demonstrating that the recovery of overbending parts can be realized without the need for molds.

**Figure 12:**
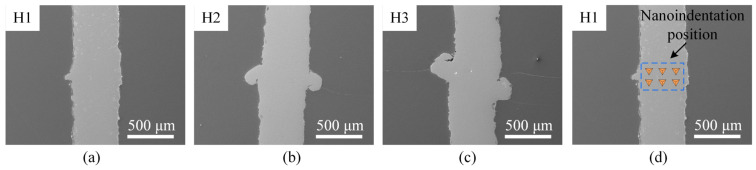
SEM morphologies of different laser bending recovery parts and the nanoindentation locations (as an example for Case H1): (**a**) Case H1, (**b**) Case H2, (**c**) Case H3, and (**d**) nanoindentation test locations for Case H1.

**Figure 13:**
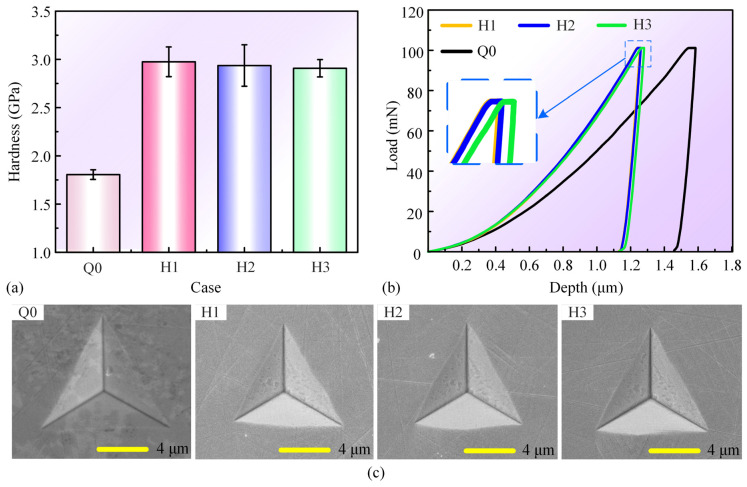
(**a**) Statistical results of hardness, (**b**) load-depth curves, and (**c**) SEM images of residual indentations on the original surface and surfaces obtained under different experimental cases.

Figure 12a–c illustrates the microscopic morphologies of the cross-section of the bending region of the laser bending recovery samples. Upon observation, the specimen surfaces are devoid of any cracks. Material buildup is observed at the same location on both sides of the sheet due to both the top and lower surfaces of the sheet being irradiated by the pulse laser, and the larger the pre-bending angle, the more material buildup is observed. To investigate the effects of laser bending recovery on the hardness of the samples, nanoindentation tests were applied to the original surface and laser-forming recovered cross-sections, and the locations of the nanoindentation tests are shown in Figure 12d. The statistical results of the hardness and load-depth curves are shown in Figure 13a and Figure 13b, respectively. The hardness of the cross-sections of the recovered samples is approximately 3 GPa, demonstrating a 66.7% increase in hardness compared to the original surface (Case Q0). Additionally, the maximum indentation depth is also reduced from ~1.58 μm to ~1.28 μm. Figure 13c illustrates the SEM images of the residual indentation obtained at a load of 100 mN captured at the same magnification. The average length of the three sides of the residual indentation for the original surface is 11.08 μm; while the average lengths of the three sides for Case H1, H2, and H3 are 9.52 μm, 9.68 μm and 9.99 μm, respectively. Compared to the original surface, the diminished size of the residual indentation in the cross-sections of the recovered samples provides further evidence for the increase in hardness of the recovered samples [51,52].

**Figure 14:**
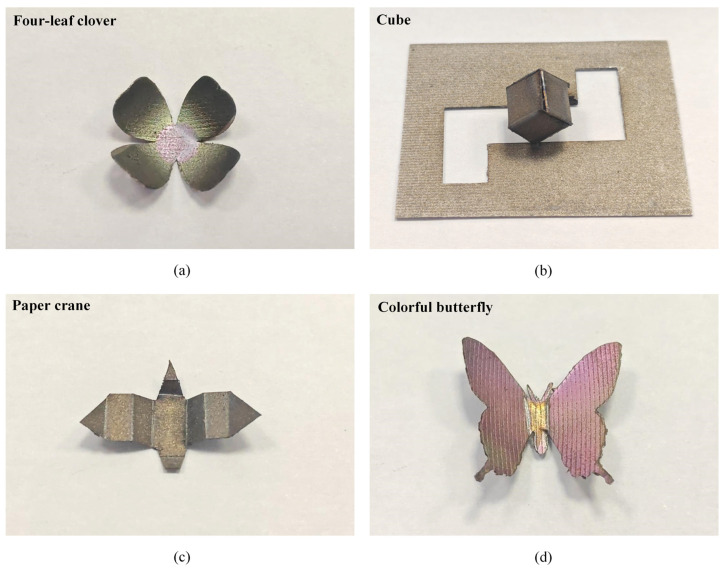
Application examples of laser bending forming for fabricating various shapes: (**a**) four-leaf clover, (**b**) cube, (**c**) paper crane, and (**d**) colorful butterfly.

Figure 14 presents some application examples for fabricating the shapes of a four-leaf clover, cube, paper crane, and colorful butterfly by combining laser bending forming with laser coloring and laser cutting techniques. The whole fabrication processes for these four shapes are completed by using the same laser system. In Figure 14a, the sheet initially undergoes rapid laser cutting to obtain a four-leaf clover shape, followed by laser bending forming using the laser multi-line scanning mode to induce curling and simultaneous coloring of the leaves. Figure 14b,c demonstrates the 3D-formed samples of a closed cube and a paper crane achieved by the laser single-line scanning mode. Additionally, Figure 14d presents a colorful butterfly. Laser irradiation is employed to color the butterfly-shaped sheet produced by laser cutting, followed by spreading wings upward using the laser single-line scanning mode. During the fabrication of the shapes depicted in Figure 14, the laser cutting parameters are configured as follows: the laser power is 40.9 W, the scanning speed is 150 mm/s, and the number of scans is 170. The parameters of the laser single-line scanning mode are set as follows: the laser power ranges from 15 W to 19.9 W, the scanning speed is from 20 mm/s to 30 mm/s, and the number of scans is fixed at 20. These examples demonstrate the application potential of laser beading forming for fabricating diverse parts.

## 4. Conclusions

The laser bending forming characteristics of CoCrFeMnNi HEA sheets, dominated by the temperature gradient mechanism, were systematically investigated. The effects of laser power, scanning speed, number of scans, and sheet size on the bending angle, forming shape, and mechanical properties were analyzed in two distinct laser scanning modes. Through experiments and analysis, the following main conclusions could be obtained.

In the laser single line scanning mode, it was found that the bending angle was affected by both the laser power and the scanning speed. At a scanning speed of 40 mm/s, when the laser power was increased from 9.6 W to 29.5 W, the bending angle of the sheets increased with an increase in laser power. At a scanning speed of 20 mm/s, when the laser power was increased from 9.6 W to 29.5 W, the bending angle increased and then decreased with the increase of the laser power, which increased from 11.6° to 47.1°, and then decreased to 20.6°. In addition, the bending angle tended to increase with the increase in the number of scans and sheet width, and decrease with the increase in sheet thickness. In laser multi-line scanning mode, it was found that the bending angle increased with the increase of laser power and decreased with the increase of scanning speed and scanning interval employed.

Under most experimental conditions, the cross-sections of the bending region exhibited smooth, homogeneous, and crack-free surfaces for both laser scanning modes. However, it was found that the higher average laser power (38.6 W) in the laser multi-line scanning mode resulted in the generation of a few holes. Therefore, in order to avoid the effect of holes, high laser power could be avoided by reducing the scanning interval when performing large angle bending. In addition, the average grain size was refined by 43% compared to the original sheet, and the hardness was significantly increased by 55.6% compared to the original surface hardness.

Through a strategic combination of laser irradiation and cooling procedures, a maximum bending angle close to 90° was obtained. J, L, and U-shaped parts were successfully fabricated. Furthermore, it was found that laser bending forming technology could be utilized to achieve recovery of overbending at different bending angles. A recovery of 100% was achieved for small-angle overbending and 91% for large-angle overbending. Lastly, it was found that various shapes such as four-leaf clovers, cubes, paper cranes, and butterflies could be fabricated by combining laser bending with laser coloring and laser cutting techniques.

## Figures and Tables

**Table 1:** Process parameters for laser single-line scanning mode.

Materials	CoCrFeMnNi HEA
Laser power, *P* (W)	9.6, 15.0, 19.9, 24.5, 29.5
Scanning speed, *v* (mm/s)	10, 20, 30, 40, 50
Number of scans, *N*	1, 5, 10, 20, 40, 60, 80, 100
Sheet thickness, *h* (mm)	0.5, 0.75, 1.00, 1.25, 1.50, 2.00
Sheet width, *w* (mm)	5, 10, 15, 20, 25

**Table 2:** Process parameters for the laser multi-line scanning mode.

Materials	CoCrFeMnNi HEA
Sheet size (mm^3^)	30 × 10 × 0.5
Scanning area size (mm^2^)	11 × 3
Laser power, *P* (W)	19.9, 24.5, 29.5, 34, 38.6
Scanning speed, *v* (mm/s)	70, 80, 90, 100, 110
Scanning interval, *L* (mm)	0.025, 0.03, 0.035, 0.04, 0.045

**Table 3:** Process parameters for different laser-formed parts in laser single-line scanning mode.

Case	Laser Power	Scanning Speed	Number of Scans	Bending Angle
L1	15.0 W	40 mm/s	20	13.7°
L2	15.0 W	40 mm/s	40	23.6°
L3	15.0 W	20 mm/s	40	43.1°
L4	24.5 W	40 mm/s	40	47.0°

**Table 4:** Process parameters for different laser-formed parts in laser multi-line scanning mode.

Case	Laser Power	Scanning Speed	Scanning Interval	Bending Angle
Q1	29.5 W	90 mm/s	0.035 mm	46.3°
Q2	29.5 W	90 mm/s	0.025 mm	78.7°
Q3	29.5 W	90 mm/s	0.045 mm	21.7°
Q4	19.9 W	90 mm/s	0.035 mm	12.9°
Q5	38.6 W	90 mm/s	0.035 mm	76.4°

**Table 5:** Laser process parameters for J, L, and U.

Part	Laser Power	Scanning Speed	Number of Scans/ Scanning Interval	Number of Processes
L	19.9 W	20 mm/s	20	7
J, U	31.8 W (1), 34.0 W (2)	90 mm/s	0.03 mm	2

**Table 6:** Process parameters for pre-bending parts.

Case	Laser Power	Scanning Speed	Number of Scans	Bending Angle
H1	9.6 W	20 mm/s	20	7.6°
H2	29.5 W	40 mm/s	20	29.3°
H3	24.5 W	40 mm/s	40	47.0°

## Data Availability

The original contributions presented in the study are included in the article, further inquiries can be directed to the corresponding author.
